# Antibiotic resistance and detection of plasmid mediated colistin resistance *mcr-1* gene among *Escherichia coli* and *Klebsiella pneumoniae* isolated from clinical samples

**DOI:** 10.1186/s13099-021-00441-5

**Published:** 2021-07-05

**Authors:** Deepa Karki, Binod Dhungel, Srijana Bhandari, Anil Kunwar, Prabhu Raj Joshi, Basudha Shrestha, Komal Raj Rijal, Prakash Ghimire, Megha Raj Banjara

**Affiliations:** 1 Central Department of Microbiology, Tribhuvan University, Kirtipur, Kathmandu, Nepal; 2 Nepalese Farming Institute, Kathmandu, Nepal; 3 Department of Microbiology, Kathmandu Model Hospital, Kathmandu, Nepal

**Keywords:** *Escherichia coli*, *Klebsiella pneumoniae*, Colistin resistance, Extended spectrum beta-lactamase, Metallo-beta-lactamase, *Klebsiella pneumoniae* carbapenemase, Multidrug resistance and plasmid-mediated mcr-1 gene

## Abstract

**Background:**

The prevalence of antimicrobial resistance (AMR) among Gram-negative bacteria is alarmingly high. Reintroduction of colistin as last resort treatment in the infections caused by drug-resistant Gram-negative bacteria has led to the emergence and spread of colistin resistance. This study was designed to determine the prevalence of drug-resistance among beta-lactamase-producing strains of *Escherichia coli* and *Klebsiella pneumoniae,* isolated from the clinical specimens received at a tertiary care centre of Kathmandu, Nepal during the period of March to August, 2019.

**Methods:**

A total of 3216 different clinical samples were processed in the Microbiology laboratory of Kathmandu Model Hospital. Gram-negative isolates (*E. coli and K. pneumoniae*) were processed for antimicrobial susceptibility test (AST) by using modified Kirby-Bauer disc diffusion method. Drug-resistant isolates were further screened for extended-spectrum beta-lactamase (ESBL), metallo-beta-lactamase (MBL), carbapenemase and *K. pneumoniae* carbapenemase (KPC) production tests. All the suspected enzyme producers were processed for phenotypic confirmatory tests. Colistin resistance was determined by minimum inhibitory concentration (MIC) using agar dilution method. Colistin resistant strains were further screened for plasmid-mediated *mcr-1* gene using conventional polymerase chain reaction (PCR).

**Results:**

Among the total samples processed, 16.4% (529/3216) samples had bacterial growth. A total of 583 bacterial isolates were recovered from 529 clinical samples. Among the total isolates, 78.0% (455/583) isolates were Gram-negative bacteria. The most predominant isolate among Gram-negatives was *E. coli* (66.4%; 302/455) and *K. pneumoniae* isolates were 9% (41/455). In AST, colistin, polymyxin B and tigecycline were the most effective antibiotics. The overall prevalence of multidrug-resistance (MDR) among both of the isolates was 58.0% (199/343). In the ESBL testing, 41.1% (n = 141) isolates were confirmed as ESBL-producers. The prevalence of ESBL-producing *E. coli* was 43% (130/302) whereas that of *K*. *pneumoniae* was 26.8% (11/41). Similarly, 12.5% (43/343) of the total isolates, 10.9% (33/302) of *E. coli* and 24.3% of (10/41) *K. pneumoniae* were resistant to carbapenem. Among 43 carbapenem resistant isolates, 30.2% (13/43) and 60.5% (26/43) were KPC and MBL-producers respectively. KPC-producers isolates of *E. coli* and *K. pneumoniae* were 33.3% (11/33) and 20% (2/10) respectively. Similarly, 63.6% (21/33) of the *E. coli* and 50% (5/10) of the *K. pneumoniae* were MBL-producers. In MIC assay, 2.2% (4/179) of *E. coli* and 10% (2/20) of *K. pneumoniae* isolates were confirmed as colistin resistant (MIC ≥ 4 µg/ml). Overall, the prevalence of colistin resistance was 3.1% (6/199) and acquisition of mcr-1 was 16.6% (3/18) among the *E. coli* isolates.

**Conclusion:**

High prevalence of drug-resistance in our study is indicative of a deteriorating situation of AMR. Moreover, significant prevalence of resistant enzymes in our study reinforces their roles in the emergence of drug resistance. Resistance to last resort drug (colistin) and the isolation of *mcr-1* indicate further urgency in infection management. Therefore, extensive surveillance, formulation and implementation of effective policies, augmentation of diagnostic facilities and incorporation of antibiotic stewardship programs can be some remedies to cope with this global crisis.

**Supplementary Information:**

The online version contains supplementary material available at 10.1186/s13099-021-00441-5.

## Background

Extensive and irrational use of antibiotics has led to the emergence and spread of antimicrobial resistance (AMR)—a condition in which pathogenic strains of bacteria develop resistance to the therapeutic antibiotics prescribed against it [1]. Realising the AMR as a global crisis, World Health Organization (WHO) and US Centres of Disease Control and Prevention (CDC) have already warned of an imminent global disaster and possibility of returning to the post-antibiotic era [2]. Gram-negative bacteria, mainly *Enterobacterales*, *Acinetobacter baumannii* and *Pseudomonas aeruginosa* are able to produce enzymes such as extended-spectrum beta-lactamases (ESBLs), AmpC beta-lactamases, carbapenemase and metallo-beta-lactamases (MBL), which enable the host bacteria to develop resistance to most classes of antibiotics in use [3]. All of these enzymes possess a similar mechanism to hydrolyse β-lactam ring of the antibiotics. ESBLs are class A β-lactamases that are responsible for resistant against oxy-imino cephalosporins (cefotaxime, ceftazidime, ceftriaxone, cefuroxime, and cefepime) and monobactams (aztreonam) [4]. Carbapenemase are the members of class A, B and D β-lactamases. Class A and D carbapenemase bring out the serine based hydrolytic mechanism while the class B carbapenemase are metallo- β-lactamases (MBL) that contain zinc based hydrolytic mechanism [5]. A novel kind of MBL, known as New Delhi metallo-β-Lactamase (NDM) possesses the ability to resist virtually all β-lactam antibiotics (except aztreonam) and carbapenems [6]. Similarly, *K. pneumoniae* carbapenemase (KPC), a derivative of carbapenemase (class A β-lactamases) also has become prominent because of their ability to inactivate carbapenems [7]. Although KPC is prevalent among *K. pneumoniae*, the enzyme has been frequently isolated from other Gram-negative bacilli [8].

Multidrug-resistant (MDR) bacteria—better known as superbugs—seriously limit the treatment options and thus are associated with increased mortalities, morbidities and economic burden [9]. On the other hand, narrowed treatment option is forcing clinicians to rely upon the “last line” drugs, primarily colistin (a polymyxin E antibiotics), which is reintroduced to counter the rapidly surging carbapenemase-producing Gram-negative bacteria [10]. Polymyxins (polymyxin B and polymyxin E) are cyclic lipopeptide discovered in the late 1940s [11] that were introduced in the treatment of infections caused by Gram-negative bacteria. However, they were no longer used due to their neuro- and nephro-toxicity and also due to the availability of comparatively ‘safer’ drugs such as beta-lactams [12]. Although their toxic effects were standstill, polymyxins were re-introduced in 1990s to counter the uncontrolled emanation of carbapenem resistant bacteria [13]. Polymyxins are now extensively used in modern clinics due to paucity of novel, effective and safer antibiotics [14]. Like with all other antibiotics, bacteria have managed to develop resistant to colistin, as a large number of studies suggest the emergence and globalization of colistin-resistance [10].

Until the first report of a variant of noble plasmid-mediated mobilized colistin resistance gene (*mcr-1*) in late 2015 in China, polymyxin (particularly, colistin) resistance was solely attributed to the regulatory changes mediated by the chromosomal genes (phoPQ, pmrAB, and mgrB) [2]. Since the first identification of *mcr-1*, several variants (from *mcr-1* to *mcr-9*) have been reported from more than 40 countries across five different continents [15]. The *mcr-1* encodes for phosphoethanolamine (pEtN) transferase enzyme (discovered in late 2015), which modifies the outer membrane lipopolysaccharides by adding pEtN to the phosphate groups in Lipid A thereby decreasing the net negative charges [2]. The resulting modification reduces the binding affinity of polymyxins to the bacterial cell wall [16, 17]. Unlike chromosomal mutation, acquisition of *mcr* is a matter of serious concern because of its potential transferability, as the gene is spread rapidly through the horizontal transfer at a higher rate than occurring through spontaneous mutation [18]. In addition, plasmids resistant to multiple classes of antibiotics can be transferred to other bacteria [19, 20]. Elevated endemicity of *mcr* genes all over the world in a short span of time is attributable to their ability to proliferate at a higher pace [21].

Since *mcr* gene was first isolated in an *E. coli* from animal sources in China, the plasmid-mediated colistin resistance may have transmitted from animals (colistin was extensively used as growth promoters for long times) to humans [22]. *E. coli* is the most prevalent species harbouring the *mcr* gene, accounting for approximately 91% of the entire load of *mcr*-positive bacteria, which is followed by *Salmonella enterica* (~ 7%) and *K. pneumoniae* (~ 2%) [23]. Higher burden of *mcr* among *S. enterica* than *K. pneumoniae* also supports the fact that the former is the food-borne pathogen and is very likely to be transmitted via food chain [24]. Moreover, these drug-resistant bacteria are isolated from humans, animals, and environments so that the perspective of ‘One Health’ has been jeopardized [25].

Implementation of effective surveillance programs and infection controls are considered as the two pillars to check the growth and spread of AMR [26]. However, in the developing countries like Nepal, circulation and co-circulation of resistant genes may go undetected, underreported and poorly characterized due to poor diagnostic facilities [27, 28]. In addition, irrational use of antibiotics among humans and animals (often as growth promoters) is putting pressure of potential outbreaks in the future [10]. Moreover, there are a limited number of studies on colistin resistance and the prevalence of resistance can vary and change over the time within and between the countries. Therefore, this study was conducted in a tertiary care center with an attempt to determine the prevalence of beta-lactamases including ESBL, MBL, KPC and colistin resistance among Gram-negative MDR pathogens. At the same time, we also aimed to explore the possible role of *mcr* genes in conferring resistance to colistin. Furthermore, antibiogram of the resistant strains to a variety of antibiotics was carried out to recognize the possible therapeutic options for combating superbugs.

## Methods

### Study design and study samples

This cross-sectional study was conducted from March to August, 2019 at Kathmandu Model Hospital, Nepal. A total of 3216 clinical samples consisting of urine (n = 1776), blood (n = 875), pus (n = 156), sputum (n = 187), body fluids (n = 88), wound swab (n = 51), tissue including femur, tibia, intestine region, and appendicular sites (n = 18), catheter and other tips (n = 12), and other samples including stool, urethral and vaginal swabs, bone, and bone marrow aspirate (n = 53) was collected and processed during the study period. Patients of all age-groups and gender who were admitted in or visiting the hospital for treatment were included in this study. All the samples with completely filled demographic information and having no visible signs of contamination were included in the study. However, others were rejected and requested for repetition, if possible.

### Sample collection and transport

All the samples were aseptically collected following the standard microbiological procedure. Individual collection procedures varied in accordance to the type of samples. Generally, samples were collected in a dry, wide-mouthed, and leak-proof container and were sent to Microbiology Department without delay. In case of unwarranted delay, clinical specimens were refrigerated at 4 to 6 °C.

### Culture, isolation and identification of bacteria

Each sample was processed by following the standard microbiological guidelines [29, 30].

#### Urine sample

Urine samples were inoculated into Cysteine Lactose Electrolyte Deficient (CLED) agar using sterile and standard calibrated loop. The plates were incubated at 37 °C overnight.

#### Blood and endotracheal and catheter tips

Blood specimen was inoculated aseptically into BHI broth at the ratio of 1: 10 and was incubated at 37 °C for 7 days and routinely inspected twice a day for at least first three days for microbial growth. Then broth from the culture bottle showing visible signs of microbial growth was sub-cultured in Blood agar (BA), MacConkey agar (MA) and Salmonella-Shigella agar (SS) and plates were incubated at 37 °C for 24 h [31].

#### Sputum and throat swab

Sputum samples were inoculated into BA, chocolate agar (CA) and MA plates. For sputum, in CA plate a 5 g$\mu$ optochin disc and a 10U bacitracin disc were added to screen *S. pneumoniae* and *H. influenzae* respectively whereas for throat swab, 0.05U bacitracin disc was added to the plate to screen *Streptococcus pyogenes*. CA and BA were incubated at 37 °C overnight in 5–10% CO_2_ environment whereas the MA plate was incubated at 37 °C in an aerobic condition [31].

#### Pus, pus swab, and wound swab

These samples were inoculated into BA and MA plates, and inoculated at 37 °C for 24 h. In case of swab, an initial inoculum was made by rubbing the swab over the media plate in order to transfer maximum number of organisms. Then the streaking was performed [31].

Body fluids**:** Body fluid samples were centrifuged before culture. The sediment after centrifugation was inoculated into BA, MA and CA plates. The BA and CA plates were incubated in 5–10% CO_2_ enriched atmosphere and MA plates were incubated aerobically at 37 °C overnight [31].

### Identification of the bacterial isolates

Following incubation, culture plates were observed for possible microbial growth. Isolates were presumably identified on the basis of Gram’s staining and colony characteristics. Further confirmation of the isolates were based on biochemical tests such as IMViC (Indole production, Methyl red test, Voges-Proskauer test and Citrate utilization), H_2_S production, catalase test, coagulase test, and oxidase test [30, 32].

### Antibiotic susceptibility test (AST)

*E. coli* and *K. pneumoniae* isolates were further subjected to *in-vitro* antibiotic susceptibility assay by using modified Kirby-Bauer disk diffusion method as recommended by Clinical Laboratory Standard Institute [33]. Nitrofurantoin (300 µg), cefotaxime (30 µg), cotrimoxazole (25 µg), cefixime (5 µg), amoxycillin (10 µg), ofloxacin (5 µg), levofloxacin (5 µg), gentamicin (10 µg), moxifloxacin (5 µg), ceftazidime (30 µg), amoxycillin/clavulanate (20/10 µg), amikacin (30 µg), ciprofloxacin (5 µg), chloramphenicol (30 µg), azithromycin (15 µg), cefoperazone/sulbactam (75/30 µg), meropenem (10 µg), imipenem (10 µg), ertapenem (10 µg), piperacillin/tazobactam (100/10 µg), doxycycline (30 µg), cefepime (5 µg), ampicillin/sulbactam (10/10 µg), polymyxin-B (100 µg), colistin (10 µg), and tigecycline (15 µg) discs were tested for susceptibility assay. In this method, broth culture of test bacteria (comparable to McFarland tube no.0.5; inoculums density 1.5 × 10^8^ bacteria/ml) was uniformly carpeted on the surface of Mueller Hinton agar (MHA). Then, antibiotics discs were placed onto the lawn culture of the test bacteriaby sterile forceps. The inoculated and seeded MHA plates were incubated at 37 ℃ for 24 h. After incubation, zone of inhibition was measured and results were interpreted as sensitive, intermediate and resistant [33]. Isolates showing resistance to at least one agent of three or more classes of antimicrobial agents were termed as multidrug-resistant (MDR) [34].

### Screening of the ESBL production

ESBL-producers were screened by using Ceftazidime (30 µg) and Cefotaxime (30 µg) in the AST. Isolates showing reduced susceptibility to one or both of these drugs with diameter of the zone of inhibition for ceftazidime ≤ 22 mm and cefotaxime ≤ 27 mm were considered as potential ESBL-producers [33]. Suspected strains were further processed using confirmatory assay.

### Confirmation of the ESBL-producers by phenotypic method

Combination disc test (CDT) as prescribed by the CLSI was used for the confirmation of ESBL-producing strains. In this method, cefotaxime (30 µg) and ceftazidime (30 µg) discs alone and in combination with clavulanic acid (10 µg) (ceftazidime plus clavulanic acid, 30/10 µg and cefotaxime plus clavulanic acid, 30/10 µg) were used. The zone of inhibition of cephalosporin disc alone was compared with their respective cephalosporin/clavulanic acid (combined) disc. An increase in zone of inhibition by ≥ 5 mm in the presence of clavulanic acid was considered as confirmed ESBL production [33].

### Screening for carbapenemase and/or KPC producers

In AST, isolates showing resistance to carbapenem drugs (imipenem 10 µg, meropenem 10 µg, and ertapenem 10 µg) were suspected as potential carbapenemase-producers [27].

### Phenotypic confirmatory test for carbapenemase and/or KPC producers

Inhibitor-based method was followed for the confirmation of carbapenemase and KPC production. In this method, combined disc test of carbapenem with and without phenyl boronic acid (PBA) was employed. An increase in the diameter of zone of inhibition by ≥ 5 mm in combined disc (carbapenem disc supplemented with PBA) than single disc (only carbapenem disc) was considered as confirmed test for carbapenem or KPC production [35, 36].

### Phenotypic confirmatory test for MBL production

Confirmation of MBL production was made by inhibition method in which Ethylene Diamine Tetra Acetic Acid (EDTA) was used as an inhibitor. Two imipenem (10 µg) discs were placed on MHA and 10 µl of 0.5 M EDTA solution was added to one of the discs to obtain the desired concentration. After overnight incubation, an increase in the diameter of zone of inhibition by 7 mm in combined disc (imipenem disc supplemented with EDTA) than single one (only imipenem disc) was considered as confirmed test for MBL production [37].

### Determination of MIC of colistin

Agar dilution method was used to determine the minimum inhibitory concentration of colistin. Different concentrations of colistin ranging from 2 µg/ml to 32 µg/ml were prepared in the agar medium. Bacterial inoculum was applied readily onto the agar surface and the plates were incubated at 37 °C upto 18 h. The MIC end point was determined as the lowest concentration of antibiotics that completely inhibits the visible growth. Isolates having a MIC of ≤ 4 μg/mL is considered colistin susceptible while MIC of > 4 μg/mL is considered colistin resistant [22, 38].

### Plasmid and genomic DNA extraction

Isolated colonies of bacteria were inoculated in Luria–Bertani (LB) broth and incubated overnight at 37 °C. After incubation, alkaline-lysis method was adopted to extract the DNA. Plasmid DNA of *E. coli* and *K. pneumoniae* was extracted by using phenol–chloroform method [39]. Extracted plasmid DNA and genomic DNA was then suspended in TE buffer and preserved at − 20 °C until further processing [39].

### PCR amplification of *mcr-1* gene

Amplification of *mcr-1* gene was carried out by conventional PCR using primers: 5′-CGGTCAGTCCGTTTGTTC-3′ as forward primer and 5′-CTTGGTCGGTCTGTAGGG-3′ as reverse primer [2]. A PCR mixture having the final volume of 25 µl (3 µl of template DNA, 0.5 µl each forward and reverse primers and 21 µl of PCR master mix) was used for the reaction mixture.

The thermal condition for amplification was initial heat activation of 95ºC for 15 min followed by 35 cycles of denaturation at 94 ℃ for 30 s; annealing at 57ºC for 90 s; extension at 72 ℃ for 90 s; and final extension at 72 ℃ for 10 min. The amplified products were subjected to gel electrophoresis (2.0% agarose gel stained with ethidium bromide) at 100 V for 60 min and visualized under UV transilluminator [22].

### Quality control during antimicrobial susceptibility and MIC assays

Each batch of media, reagents and antibiotic discs were checked for their lot number, expiry date, and proper storage. Similarly, purity plates were used to ensure the pure culture of test organisms. Control strain of *E. coli* ATCC 25922 was used during AST.

### Data analysis

All the data were entered in the worksheet of Statistical Package for Social Sciences (SPSS) software (Version 25). The results have been presented in the form of tables and figures. Chi-square (χ2) test was applied to test the association between the variables. A *p* value of < 0.05 was considered as statistically significant.

## Results

### Distribution of bacterial isolates

Among the total samples processed, 16.4% (529/3216) had bacterial growth.. Higher percent of bacterial isolates were obtained from urine samples (304/529; 57.5%) followed by pus (81/529; 15.3%) and blood samples (49/529; 9.3%). More than half of the isolates (300/529; 56.7%) were recovered from the female population. The bacterial infection was in high percentage in the age group 16–45 years (260/529; 49.1%) followed by the older age group above 60 years (158/529; 29.9%) (Table 1).

**Table 1:** Demographic and clinical character of patients attending at Model Hospital, Kathmandu

Character	No. of total samples	Culture positive		p-value
		Number	%	
Clinical specimens				
Urine	1776	304	57.5	
Blood	875	49	9.3	
Pus	156	81	15.3	
Sputum	187	24	4.5	
Body fluids	88	6	1.1	
Wound swab	51	34	6.4	
Tissue	18	9	1.7	
Catheter tips and other tips	12	9	1.7	
Other samples	53	13	2.5	
Total	3216	529		
Gender				
Male	1933	229	43.3	0.0
Female	1283	300	56.7	
Age group (years)				
0–15	299	18	3.4	0.01
16–45	1718	260	49.1	
46–60	482	93	17.6	
> 60	717	158	29.9	

**Fig. 1:**
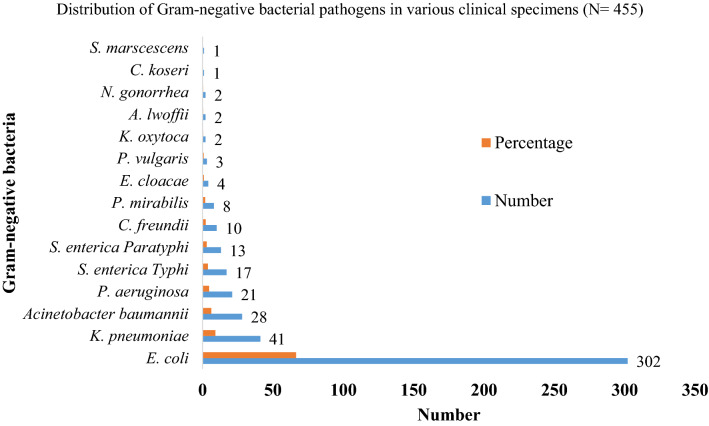
Distribution pattern of Gram-negative bacterial pathogens

Out of 529 culture positive specimens, 50 samples showed polymicrobial growth and 473 samples showed monomicrobial growth. Due to polymicrobial infections, 583 isolates were recovered from 529 samples. Among total isolates, 78.0% (455/583) were Gram-negative bacteria. The most predominant isolate among Gram-negatives was *E. coli* (66.4%; 302/455) followed by *K. pneumoniae* (9.0%; 41/455) and *Acinetobacter calcoaceticus baumannii complex* (6.2%; 28/455), *Pseudomonas aeruginosa* (4.6%; 21/455), *Salmonella enterica* Typhi (3.7%, 17/455) and *Salmonella enterica* Paratyphi (2.9%; 13/455). Small portion of the isolates included other bacteria such as *Citrobacter koseri*, *Serratia marscescens*, *Acinetobacter lwoffii, Klebsiella oxytoca,* and *Neisseria gonorrheae*. Distribution of the isolates is depicted in the Fig. 1.

In this way, 9.4% (301/3216) and 1.3% (35/3216) isolates of *E. coli* and *K. pneumoniae* respectively were obtained from the total sample processed. Majority of these isolates were obtained from urine (78.9%; 265/336) followed by pus (9.2%; 30/336) and wound swab (3.9%; 13/336). Respectively, 240 and 25 isolates of *E. coli* and *K. pneumoniae* were from urine samples. Similarly, large number of samples processed and the isolates recovered were predominantly obtained from female patients of the age group of 16–45 years (Additional file 1; Table 2).

**Table 2:** Distribution of *E. coli* and *K. pneumoniae* according to sex, age and type of samples

Character	*E. coli*	*K. pneumoniae*	Total	p-value
	N (%)	N (%)	N (%)	
Gender				
Male	93 (30.9)	13 (37.1)	106 (31.5)	0.45
Female	208 (69.1)	22 (62.9)	230 (68.5)	
Age group in years				
0–15	8 (2.7)	1 (2.9)	9 (2.7)	0.08
16–45	142 (47.2)	6 (17.1)	148 (44)	
46–59	57 (18.9)	11 (31.4)	68 (20.2)	
> 60	94 (31.2)	17 (48.6)	111 (33.0)	
Total	301	35	336	
Clinical specimens				
Urine	240 (79.7)	25 (71.4)	265 (78.9)	0.17
Wound swab	12 (4.0)	1 (2.9)	13 (3.9)	
Pus	28 (9.3)	3 (8.6)	30 (9.2)	
Blood	6 (2.0)	0 (0)	6 (1.8)	
Sputum	6 (2.0)	4 (11.4)	10 (3.0)	
Various tips	3 (1.0)	0 (0)	3 (0.9)	
Fluids	4 (1.3)	1 (2.9)	5 (1.5)	
Foley's Catheter tube	1 (0.3)	0 (0)	1 (0.3)	
Tissues	1 (0.3)	1 (2.9)	2 (0.6)	

### Antibiotic susceptibility pattern of *E. coli* and *K. pneumoniae*

All of the tested isolates of *E. coli* and *K. pneumoniae* were susceptible to colistin, polymixin B and tigecycline. *E. coli* isolates were also susceptible to antibiotics nitrofurantoin (93.5%; only for uropathogens), gentamycin (87.8%) and amikacin (89.8%). However, all of the tested *E. coli* isolates were resistant to azithromycin. Similarly, *K. pneumoniae* isolates were resistant to amoxycillin and azithromycin. Resistance of *K. pneumoniae* towards cephalosporin antibiotics include: cefepime (57.1%), ceftazidime (55.6%) and cefotaxime (51.2%). None of the isolates was resistant to ciprofloxacin, colistin, polymyxin-B and tigecycline (Table 3).

**Table 3:** Antibiotic susceptibility pattern of *E. coli* and *K. pneumoniae*

Organism/antibiotics used	*E. coli*			*K. pneumoniae*		
	Sensitive	Resistant	Total	Sensitive	Resistant	Total
	N (%)	N (%)		N (%)	N (%)	
1st line antibiotics						
Nitrofurantoin	230 (93.5)	16 (6.5)	246	14 (53.8)	12 (46.1)	26
Cefotaxime	130 (43.1)	172 (56.9)	302	20 (48.8)	21 (51.2)	41
Cotrimoxazole	156 (51.7)	146 (48.3)	302	29 (70.7)	12 (29.3)	41
Cefixime	122 (47.3)	136 (52.7)	258	18 (60.0)	12 (40.0)	30
Amoxycillin	81 (26.8)	221 (73.2)	302	0 (0.0)	41 (100)	41
Ofloxacin	153 (51.3)	145 (48.7)	298	25 (67.6)	12 (32.4)	37
Levofloxacin	154 (51.0)	148 (49.0)	302	29 (70.7)	12 (29.3)	41
Gentamycin	265 (87.8)	37 (12.2)	302	31 (75.6)	10 (24.4)	41
Moxifloxacin	129 (52.7)	116 (47.3)	245	20 (76.9)	6 (23.1)	26
Ceftazidime	71 (48.3)	76 (51.7)	147	8 (44.4)	10 (55.6)	18
2nd line antibiotics						
Amoxycillin/Clavulanate	63 (40.1)	94 (59.9)	157	8 (38.1)	13 (61.9)	21
Amikacin	141 (89.8)	16 (10.2)	157	11 (52.4)	10 (47.6)	21
Ciprofloxacin	5 (45.5)	6 (54.5)	11	4 (100)	0 (0.0)	4
Chloramphenicol	25 (73.5)	9 (26.5)	34	3 (37.5)	5 (62.5)	8
Azithromycin	0 (0.0)	5 (100)	5	0 (0.0)	4 (100)	4
Cefoperazone/Sulbactam	49 (36.8)	84 (63.2)	133	2 (14.3)	12 (85.7)	14
Meropenem	100 (75.2)	33 (24.8)	133	4 (28.6)	10 (71.4)	14
Imipenem	100 (75.2)	33 (24.8)	133	4 (28.6)	10 (71.4)	14
Ertapenem	100 (75.2)	33 (24.8)	133	4 (28.6)	10 (71.4)	14
Piperacillin/Tazobactam	98 (73.7)	35 (26.3)	133	4 (28.6)	10 (71.4)	14
Doxycycline	41 (30.8)	92 (69.2)	133	3 (21.4)	11 (78.6)	14
Cefepime	76 (57.1)	57 (42.9)	133	6 (42.9)	8(57.1)	14
Ampicillin/Sulbactam	16 (51.6)	15 (48.4)	31	1 (14.3)	6 (85.7)	7
3rd line antibiotics						
Polymyxin-B	32 (100)	0 (0.0)	32	9 (100)	0 (0)	9
Colistin	32 (100)	0 (0.0)	32	9 (100)	0 (0)	9
Tigecycline	32 (100)	0 (0.0)	32	9 (100)	0 (0)	9

### Prevalence of MDR, ESBL producing bacteria, and carbapenem resistant bacteria

The overall prevalence of MDR among both of the isolates was 58.0% (199/343). Individually, 59.2% (179/302) of the total *E. coli* isolates and 48.7% (20/41) of the *K. pneumoniae* isolates were reported as MDR (Table 4).

**Table 4:** Distribution of MDR, ESBL and Carbapenem resistant in *E. coli* and *K. pneumoniae* in relation to gender, age and clinical specimens (n = 336)

Character	*MDR*	p-value	*ESBL producers*	p-value	Carbapenem resistant	p-value
	N (%)		N (%)		N (%)	
Gender						
Male	66 (34.4)	0.19	39 (28.7)	0.09	21 (55.3)	0.02
Female	126 (65.6)		97 (71.3)		17 (44.7)	
Total	192		136		38	
Age group in years						
0–15	8 (4.2)	0.00	4 (2.9)	0.001	2 (5.3)	0.00
16–45	64 (33.3)		45 (33.1)		12 (31.6)	
46–59	51 (26.6)		38 (27.9)		13 (34.2)	
> 60	69 (35.9)		49 (36.0)		11 (28.9)	
Total	192		136		38	
Clinical specimens						
Urine	143 (75.0)	0.16	105 (77.2)	0.019	25 (65.8)	0.25
Wound swab	8 (4.0)		6 (4.4)		3 (7.9)	
Pus	25 (13)		16 (11.8)		7 (18.4)	
Blood	2 (1.0)		2 (1.4)			
Sputum	5 (2.5)		2(1.4)			
Various tips	2 (1.0)				1 (2.6)	
Fluids	4 (2.0)		3 (2.1)		1 (2.6)	
Foley's Catheter tube	1 (0.5)		1 (0.7)			
Tissues	2 (1.0)		1 (0.7)		1 (2.6)	
Organisms						
*E. coli*	179 (89.9)	0.03	130 (92.1)	0.033	33 (76.8)	0.01
*K. pneumoniae*	20 (10.1)		11 (7.9)		10 (23.2)	

Of 343 bacterial isolates, 41.1% (n = 141) isolates were confirmed as ESBL-producers. The prevalence of ESBL-producing *E. coli* was 43% (130/302) whereas that of *K*. *pneumoniae* was 26.8% (11/41) (Table 4).

The prevalence of carbapenem resistance among both the isolates was 12.5% (43/343). Comparatively, higher percentage of carbapenem resistance was documented among *E. coli* (10.9%; 33/302) than *K. pneumoniae* (24.3%; 10/41) (Table 4).

### Distribution of KPC and MBL among carbapenem resistant *E. coli* and *K. pneumoniae*

**Fig. 2:**
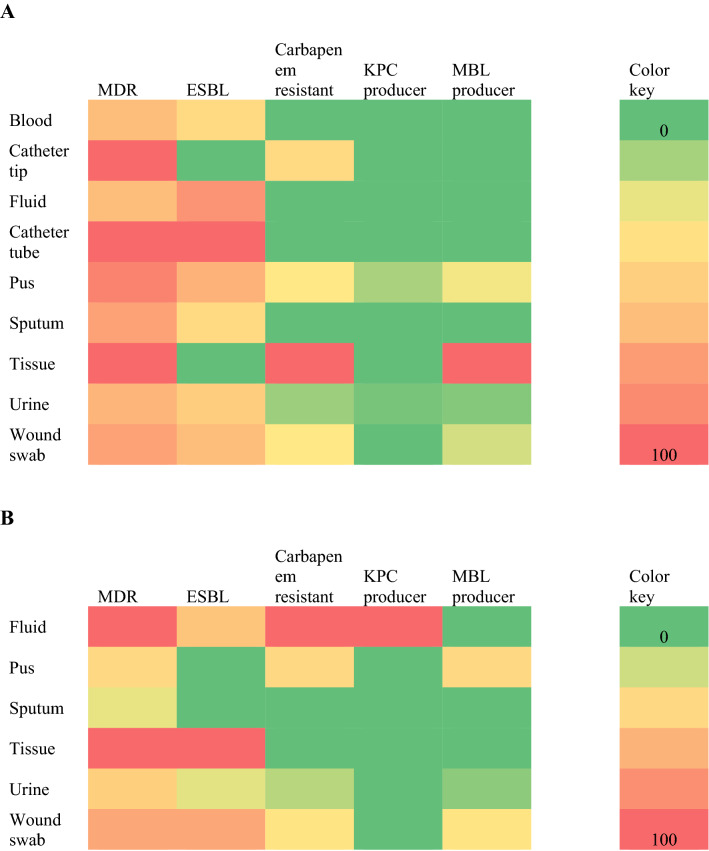
Heat map showing the distribution of MDR, carbapenem resistant and ESBL, KPC and MBL producers among *E. coli* (**A**) and *K. pneumoniae* (**B**) in different clinical samples. The red box indicates more prevalent in the clinical specimen compared to green box. The colour gradient from red to green displays a linear scale of the percent distribution from high to low as a measure of the MDR, carbapenem resistant and ESBL, KPC and MBL producers

Among 43 carbapenem resistant isolates, 30.2% (13/43) and 60.5% (26/43) were KPC and MBL-producers respectively. Both KPC and MBL production was reported higher among *E. coli* in comparison to *K. pneumoniae*. 33.3% (11/33) and 20% (2/10) of the isolates of *E. coli* and *K. pneumoniae* were KPC-producers respectively. Similarly, 63.6% (21/33) and 50% (5/10) of the *E. coli* and *K. pneumoniae* were MBL-producers, respectively (Fig. 2).

### Determination of MIC and colistin resistant *E. coli* and *K. pneumoniae* isolates

In MIC assay, 2.2% (4/179) of *E. coli* and 10% (2/20) of *K. pneumoniae* isolates were confirmed as colistin resistant (MIC ≥ 4 µg/ml). Overall, the prevalence of colistin resistance among the tested isolates was 3.01% (6/199) (Table 5). MIC of colistin-resistant isolates of *E. coli* and *K. pneumoniae* ranged from ≤ 4 µg/ml to 8 µg/ml and from ≤ 8 µg/ml to 16 µg/ml respectively.

**Table 5:** Characteristics of colistin resistant strains

Characteristics	*Escherichia coli*	*Klebsiella pneumoniae*
Number of isolates tested	179	20
Colistin resistant by MIC determination	4 (2.2%)	2 (10%)
Specimen types of colistin resistant bacteria	Urine for all isolates	Urine for all isolates
In-patient/out-patient	Inpatient- 2 Outpatient- 2	Inpatient- 2
Colistin resistance (MIC µg/ml)	4 µg/ml-3 8 µg/ml-1	8 µg/ml-1 16 µg/ml-1
*mcr-1* plasmid detected in colistin resistant	3	0
*mcr-1* chromosome detected in colistin resistant	2	0
*mcr-1* both plasmid and chromosome detected in colistin resistant	2	0
ESBL producer	2	2
Carbapenem resistance isolates	1	1
MBL producer	1	1
KPC producer	0	0
Isolates with MIC break-point (2 µg/ml) (colistin sensitive)	18	0
*mcr-1* plasmid detected in colistin sensitive	5	0
*mcr-1* chromosome detected in colistin sensitive	12	0
*mcr-1* both plasmid and chromosome detected in colistin sensitive	3	0

### PCR amplification of *mcr-1* in colistin resistant and sensitive *E. coli* and *K. pneumoniae*

Out of four phenotypically colistin-resistant *E. coli*, 3 (75.0%) and 2 (50%) of them harboured plasmid-mediated and chromosomal *mcr-1* gene respectively (Table 5). In contrast, none of the phenotypically colistin resistant *K. pneumoniae* harboured *mcr-1* gene.

Among the 193 colistin sensitive MDR isolates, PCR amplification for mcr-1 was performed with 18 *E. coli* isolates exhibiting an MIC breakpoint of 2 μg/ml. Out of 18 MDR *E. coli* with MIC 2 µg/ml, 16.6% (3/18) and 61.1% (11/18) harboured plasmid-mediated and chromosomal *mcr-1* genes respectively (Table 5).

### Antibiotic resistance profiles of colistin resistant and sensitive isolates

**Fig. 3:**
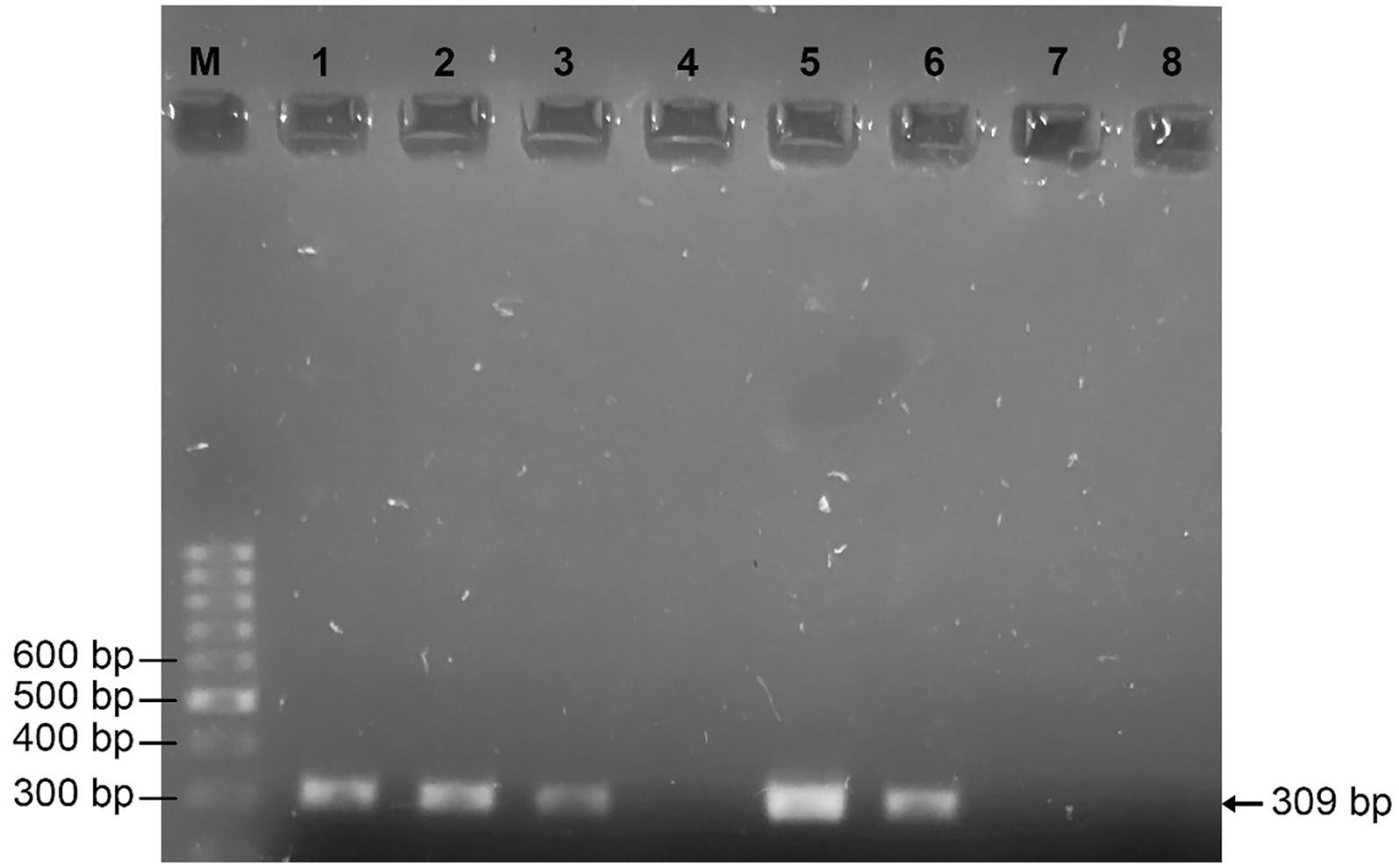
PCR amplification of *mcr-1* gene in colistin resistant *E. coli* (Lane M, DNA size marker (100-1000 bp); Lane 1: positive control (plasmid template), Lane 2/3/5/6: *mcr-1* positive isolates (plasmid template) (309 bp), Lane 4: negative control (no template) and Lane 7/8 *mcr-1* negative isolates

Antibiotic resistance profiles of colistin resistance *E. coli* and *K. pneumoniae* were determined. Colistin resistant 100% *E. coli* isolates were resistant to cefotaxime, cefixime, amoxycillin, ofloxacin, levofloxacin, amoxycillin/clavulanate, and doxycycline where as colistin resistant 100% *K. pneumoniae* isolates were resistant to nitrofurantoin, cefotaxime, cefixime, amoxycillin, ofloxacin, and levofloxacin (Fig. 3, Table 6).

**Table 6:** Antibiotic resistance profiles of colistin resistant and sensitive isolates

Antibiotics	Colistin resistant *E. coli* (N = 4) (%)	Colistin sensitive *E. coli* (N = 175) (%)	Colistin resistant *K. pneumoniae* (N = 2) (%)	Colistin sensitive *K. pneumoniae* (N = 18) (%)
NIT	0	5.7	100	33.3
CTX	100	79.4	100	100
COT	50	53.7	50	55.6
CFM	100	62.3	100	55.6
AMX	100	88	100	100
OF	100	56	100	55.6
LE	100	57.1	100	55.6
GEN	25	18.3	50	50
CAZ	50	60.6	100	66.7
AMC	100	42.8	100	61.1
AK	25	7.4	50	50
CFS	75	37.1	100	55.6
MRP	25	14.9	50	50
IMP	25	14.9	50	50
ETP	25	14.9	50	50
PIT	25	14.9	50	50
DOX	100	44.6	100	50
CX	25	16.6	100	16.7
CPM	100	50.3	100	55.6
A/S	0	6.9	0	33.3
TGC	0	0	0	0

Nitrofurantoin (NIT); cefotaxime (CTX); cotrimoxazole (COT); cefixime (CFM); amoxycillin (AMX); ofloxacin (OF); levofloxacin (LE); gentamycin (GEN); ceftazidime (CAZ); amoxycillin/clavulanate (AMC); amikacin (AK), cefoperazone/sulbactam (CFS); meropenem (MRP); imipenem (IMP); ertapenem (ETP); piperacillin/tazobactam (PIT); doxycycline (DOX); cefepime (CPM); ampicillin/sulbactam (A/S); tigecycline (TGC)

## Discussion

Drug-resistance, especially pan-drug resistance and MDR is emerging as a major challenge in the treatment of infections caused by Gram-negative bacteria. As surveillance of AMR and early response to the infection control are crucial steps to address the issues, this study also aimed to determine the status of MDR among *E. coli* and *K. pneumoniae* isolates and to investigate possible acquisition of *mcr-1* in colistin resistant isolates. In this study, most of the isolates were resistant to commonly prescribed broad-spectrum antibiotics. In addition, prevalence of colistin resistance and acquisition of *mcr-1* among the drug-resistant isolates was also observed in this study.

In this study, 16.4% (529/3216) specimens showed bacterial growth in which prevalence of Gram-negative bacteria from different clinical specimens was much higher than the Gram-positives. This finding concords with previous studies reported from Nepal [9, 10, 40–44]. Higher prevalence of *E. coli* in comparison to other species could be due to being normal flora of human gut which is highly opportunistic in immunocompromised patients. When *E. coli* reaches out to the tissues other than its common site, it serves as an opportunistic pathogen. A number of virulence factors encoded by pathogenic strains of *E. coli* enable them to colonize the human body in spite of effective host defence [45].

In this study, all of the isolates of *E. coli* and *K. pnemoniae* were susceptible to colistin, polymyxin B and tigecycline, which is comparable to some previous findings [46, 47]. These classes of antibiotics can be effective drugs in the management of Gram-negatives. Conversely, all of the isolates were resistant towards azithromycin. Previous exposure of the isolates to these antibiotics as well as the state of resistance genes of corresponding antibiotics may be the reasons for their susceptibility patterns [48].

In this study, increased resistance to third-generation cephalosporins was observed, as more than half of the isolates were non-susceptible to those drugs. Similar findings have been reported by some previous studies [47, 49, 50]. Higher rate of resistance to cephalosporins can be attributable to their irrational prescription and uses [51].

Resistance rate of *E. coli* to fluoroquinolones in this study ranged from 47 to 55% which are in agreement with earlier studies from Nepal [52, 53] and India [54]. Resistance to fluroquinolones among MDR Gram-negative bacteria is common and is expected to sustain and perhaps accelerate even if other antibiotics are used [55]. The prevalence of fluroquinolone resistance is related to the intensity of antibiotics used, which may reduce the efficacy of drug in a progressive manner [56].

In this study, almost one fourth of *E. coli* isolates were resistant to carbapenem antibiotics. The resistance rate towards these antibiotics ranged from < 3.0% to 21.0% in some of the previous studies from Nepal [50, 52, 53] whereas 100% sensitivity towards imipenem was reported in some other studies [47, 57]. Production of beta-lactamase enzymes and the upregulation of efflux pump are suggested as the reasons for reduced susceptibility [58]. However, comparatively low resistance to carbapenem antibiotics reported in this study could be due to the lower use of these antibiotics in the treatment of infections [59].

In AST assay of *K. pneumoniae*, two-third of the isolates were susceptible to fluoroquinolones and gentamicin. This finding is similar to another study reported from Nepal [60]. However, some other studies have reported lower sensitivity rates (less than 50.0%) [57, 61]. This variation may be due to the difference in the specimens included in the study as well as the exposure of isolates towards the antibiotics. All of the *K. pneumoniae* isolates were resistant to amoxicillin. Similar findings were reported in other studies [52, 60]. In addition, reduced sensitivity towards cephalosporin and carbapenem antibiotics was observed in this study. High resistant rate towards cephalosporins was also reported in previous studies [50, 52, 62, 63]. Multiple factors such as extensive use of drugs, production of beta-lactamases, or efflux pumps (which actively pump out these antibiotics) are attributable to the rise in the resistance against carbapenems [26, 64].

Among the total (343) isolates of *E. coli* and *K. pneumoniae*, more than half (58.0%) were MDR. MDR strains were predominant among the isolates of *E. coli* in comparison to *K. pneumoniae*. This result was in consistent with previous findings which also reported the rate of MDR in a range of 41.0%–67.7% [9, 20, 28, 52, 60] while lower than some other findings [8, 10]. In this study, the prevalence of MDR *E. coli* and *K. pneumoniae* was 59.3% and 48.8% respectively. Common risk factors associated with development of MDR are poor hygiene, misuse of antibiotics and absence of antimicrobial surveillance program [65, 66]. Higher rate of antibiotic resistance among *E. coli* and *Klebsiella* spp*.* is associated with their ability to produce different kinds of β-lactamases primarily ESBL, AmpC and MBL, and carriage of resistance trait for quinolones and aminoglycosides in the plasmid [67]. In several hospitals in Nepal, the antibiotics used for the treatment of infected patients are effective in curing only a half of the cases whereas other half of the treatment course shows no response [68]. In addition, development of partial resistance by bacteria, most antibiotics intended to cure people are becoming less effective which might also be the reason of increasing prevalence of MDR reported in this study [26].

In this study, the prevalence of ESBL producing strains was found to be 41.1% among Gram-negative isolates. This result is comparable to some previous reports from Nepal [52, 69]. The prevalence of ESBL production was reported low in other studies [70–72]. The difference in the prevalence of ESBL production can be partially due to geographical variations, type of specimens processed, and local practices of antibiotics prescription and use [73].

In our study, among 343 isolates of *E. coli* and *K. pneumoniae,* 12.5% were resistant to carbapenem. In earlier study reported from Kathmandu Model Hospital, the prevalence of carbapenem resistant ranged from 4.5% to 20.0% among the members of Enterobacteriaceae [74]. However, higher rate of carbapenem resistant among Enterobacteriaceae was reported in other studies [75, 76]. The difference in utilization of carbapenem antibiotics to treat infections in different study settings may be responsible for these variations [75].

Carbapenem resistant isolates were subjected to KPC and MBL production test phenotypically. In this assay, 30.2% and 60.5% isolates were KPC and MBL-producers respectively. Our findings are comparable to a previous finding [77]. The prevalence of KPC production in *E. coli* and *K. pneumoniae* was 33.3% and 20.0% respectively in our study. Similarly, MBL production was reported 63.6% in *E. coli* and 50.0% in *K. pneumoniae.* In a previous study, KPC production in *E. coli* was reported as 14.4% and 7.1% in *K. pneumoniae* [8]. Accordingly, another study reported comparatively lower incidence of MBL producing-*E. coli* and *K. pneumoniae* in different clinical samples in Central Nepal [78]. Similarly, lower rate of MBL (9.0%) and KPC (6.5%) production was reported in *E. coli* [79]. Another study from Iran reported 80.5% of *K. pneumoniae* isolates as KPC-producers [80]. This study revealed higher prevalence of MBL and KPC production in *E. coli* and *K. pneumoniae* which may be due to dissemination of plasmid encoded carbapenem resistance genes [59].

This study reported comparatively low prevalence (3.0%) of colistin resistant *E. coli* and *K. pneumoniae* isolates (3.0%). Similar result was reported in previous findings of 0.3% in Switzerland [81], 0.7% in Spain [82] and in a previous report of global antimicrobial surveillance programs [83, 84]. Low prevalence of the resistant isolates in our study may be attributable to the presence of low number of *mcr-1* positive bacteria and lesser use of colistin use for the treatment of community acquired infections [81]. However, other studies from India [85] and Thailand [86] reported the prevalence rate of colistin resistant as high as 32.0% and 71.3% respectively. In this study, rates of colistin resistance were different among the bacterial species. Higher prevalence of colistin resistance was reported in *K. pneumoniae* (10.0%) when compared to *E. coli* (2.2%). Different studies also showed that higher prevalence of colistin resistant *K. pneumoniae* than *E. coli* [87, 88]. The main risk factor for the development of colistin resistance is related to the extensive and irrational use of colistin in antimicrobial therapy [89].

MIC range of colistin for *E. coli* ranged from ≤ 2 µg/ml to 8 µg/ml which was lesser than that of *K. pneumoniae* (≤ 2 µg/ml to 16 µg/ml). This finding is in agreement with a previous study from China [90]. MIC range of *mcr-1* positive *Enterobacteriaceae* typically have a moderate level 4–16 mg/l of colistin resistant strains [91]. MIC of colistin resistant isolates carrying *mcr-1* was lower in this study. Exceptionally, MIC range of colistin in colistin resistant *K. pneumoniae* without *mcr-1* was high in this study. This result suggests that colistin resistance in *K. pneumoniae* might be associated with chromosomal mutations in *mgrB, phoP/phoQ, pmrA, pmrB, pmrC* and *crrABC* [92]. High MIC may also be due to strong selective pressure in the isolates. These strains may carry another variant of *mcr* gene [93].

## Strength and limitations

This is the first study from Nepal which also determined the prevalence of *mcr-1* among colistin susceptible isolates. In addition, this study is one among a handful of studies that attempted to investigate the role of the beta-lactamases (ESBL, MBL, and carbapenemase) and acquisition of *mcr-1* among Gram-negative isolates. The findings of this study can be an important reference tool for policy makers and clinicians which can ameliorate the practice of antibiotic prescription and use in the country. However, this study suffers from some limitations. Firstly, this study was conducted among small population of a tertiary care centre so that the reported rates cover a limited geographical region and may not reflect overall picture of epidemiology across the country. Secondly, this study tested the acquisition of only one variant (*mcr-1*) of *mcr* gene; isolation and characterization of all variants (*mcr-1 to mcr-9*) is suggested in future studies to predict the role of genes in conferring resistance. In addition, this study could not predict the origin and possible transferability of resistant strains. The insertion sequence IS*Apl1* plays an important role in the mobilization of this *mcr-1* gene. However, in this study only *mcr-1* gene was analysed. Therefore, further molecular analysis can better predict other possible mechanisms responsible for colistin resistance and role of insertion sequence in dissemination of this gene.

## Conclusion

High burden of MDR strains in our study could be due to the pervasive and irrational practices of antibiotic prescription and use, as half of the Gram-negative isolates were found as drug-resistant. Moreover, the presence of colistin resistance and acquisition of *mcr-1* among clinical isolates warrants an imminent threat of no antibiotic era. Therefore, prompt action is recommended for proper infection control. Augmentation of diagnostic facilities, AMR surveillance, and antibiotic stewardship programs can be some effective measures to address the problem of drug-resistance in the country.

## Supplementary Information

**Additional file 1: Table S1.** Characteristics of infected patients and samples.

## Data Availability

The datasets used and/or analyzed during the current study available from the corresponding author on reasonable request.

## Abbreviations

AMR: Antimicrobial Resistance
AST: Antimicrobial susceptibility testing
CLSI: Clinical and Laboratory Standard Institute
CPE: Cytopathic effect
CRE: Carbapenem resistant *E. coli*
ESBL: Extended spectrum β-lactamase
GNB: Gram-negative bacteria
ICU: Intensive care unit
kDa: Kilo-Dalton
LPS: Lipopolysaccharide
*mcr-1*: Mobilized colistin resistance
MDR: Multidrug resistance
MIC: Minimum inhibitory concentration
ORF: Open reading frame
PCR: Polymerase chain reaction
UTI: Urinary tract infection
WHO: World Health Organization
WGS: Whole Genome Sequencing
