# Supplementary material for: Antibiotic resistance and detection of plasmid mediated colistin resistance mcr-1 gene among Escherichia coli and Klebsiella pneumoniae isolated from clinical samples
Source: Gut Pathog. 2021 Jul 5;13:45. doi: 10.1186/s13099-021-00441-5 (PMC8256586; doi:10.1186/s13099-021-00441-5)
Supplement: Supplementary file 1 — Additional file 1: Table S1. Characteristics of infected patients and samples. [file 13099_2021_441_MOESM1_ESM.docx]

Table S1: Characteristics of infected patients and samples

| **Particulars** | ***E. coli*** | | | ***K. pneumoniae*** | | | **Total isolates** | | |
| --- | --- | --- | --- | --- | --- | --- | --- | --- | --- |
|  | **Male**  **n (%)** | **Female n (%)** | **Total n (%)** | **Male**  **n (%)** | **Female n (%)** | **Total n (%)** | **Male**  **n (%)** | **Female n (%)** | **Total**  **n (%)** |
| *Age group (years)* |  |  |  |  |  |  |  |  |  |
| Less than or equal to 15 | 6 (6.5) | 2 (1.0) | 8 (2.7) | 0 | 1 (4.5) | 1 (2.9) | 6 (5.7) | 3 (1.3) | 9 (2.7) |
| 16-45 | 37 (39.8) | 105 (50.5) | 142 (47.2) | 0 | 6 (27.3) | 6 (17.1) | 37 (34.9) | 111 (48.3) | 148 (44.1) |
| 46-59 | 17 (18.3) | 40 (19.2) | 57 (18.9) | 5 (38.5) | 6 (27.3) | 11 (31.4) | 22 (20.8) | 46 (20.0) | 68 (20.2) |
| 60 or more | 33 (35.5) | 61 (29.3) | 94 (31.2) | 8 (61.5) | 9 (40.9) | 17 (48.6) | 41 (38.7) | 70 (30.4) | 111 (33.0) |
| Total | 93 | 208 | 301 | 13 | 22 | 35 | 106 | 230 | 336 |
| *Type of specimens* |  |  |  |  |  |  |  |  |  |
| Blood | 2 (2.2) | 4 (1.9) | 6 (2.0) | 0 | 0 | 0 | 2 (1.9) | 4 (1.7) | 6 (1.8) |
| Catheter tips | 1 (1.1) | 2 (1.0) | 3 (1.0) | 0 | 0 | 0 | 1 (0.9) | 2 (0.8) | 3 (0.9) |
| Fluids | 0 | 4 (2.0) | 4 (1.3) | 1 (7.7) | 0 | 1 (2.9) | 1 (0.9) | 4 (1.7) | 5 (1.5) |
| Catheter tube | 0 | 1 (0.5) | 1 (0.3) | 0 | 0 | 0 | 0 | 1 (0.4) | 1 (0.3) |
| Pus | 15 (16.1) | 13 (6.3) | 28 (9.3) | 2 (15.4) | 1 (4.5) | 3 (8.6) | 17 (16.0) | 14 (6.1) | 31 (9.2) |
| Sputum | 4 (4.3) | 2 (1.0) | 6 (2.0) | 2 (15.4) | 2 (9.1) | 4 (11.4) | 6 (5.7) | 4 (1.7) | 10 (3.0) |
| Tissue | 1 (1.1) | 0 | 1 (0.3) | 1 (7.7) | 0 | 1 (2.9) | 2 (1.9) | 0 | 2 (0.6) |
| Urine | 63 (67.7) | 177 (85.1) | 240 (79.7) | 7 (53.8) | 18 (81.8) | 25 (71.4) | 70 (66.0) | 195 (84.8) | 265 (78.9) |
| Wound swab | 7 (7.5) | 5 (2.4) | 12 (4.0) | 0 | 1 (4.5) | 1 (2.9) | 7 (6.6) | 6 (2.6) | 13 (3.8) |
| Total | 93 | 208 | 301 | 13 | 22 | 35 | 106 | 230 | 336 |
